# Flow as an Embodied State. Informed Awareness of Slackline Walking

**DOI:** 10.3389/fpsyg.2019.02993

**Published:** 2020-01-10

**Authors:** Lluc Montull, Pablo Vázquez, Lluís Rocas, Robert Hristovski, Natàlia Balagué

**Affiliations:** ^1^Complex Systems in Sport Research Group, Institut Nacional d’Educació Física de Catalunya (INEFC), Universitat de Barcelona, Barcelona, Spain; ^2^Complex Systems in Sport Research Group, Faculty of Physical Education, Sport and Health, Ss. Cyril and Methodius University in Skopje, Skopje, Macedonia

**Keywords:** radical embodiment, informed awareness, ecological dynamics, skill, stability, slackline

## Abstract

Flow during exercise has been theorized and studied solely through subjective-retrospective methods as a “scull bound” construct. Recent advances of the radical embodied perspectives on conscious mind and cognition pose challenges to such understanding, particularly because flow during exercise is associated with properties of performer’s movement behavior. In this paper we use the concept of informed awareness to reconceptualize flow experience as a property of the performer-environment coupling, and study it during a slackline walking task. To empirically check the possible relatedness of the behavior-experience complementary pair, two measures were considered. The experiential realm was quantified by the flow short scale and the behavioral realm by the Hurst (H) exponent obtained through accelerometry time series of the legs and the center of body mass (CoM). In order to obtain a coarse-grained insight about the degree of co-varying within the perception-action flow of performers, we conducted correlational and multiple regression analyses. Measures of behavioral variables (H exponents of the dominant, subdominant leg and the CoM, were treated as explanatory, and the flow scale and its subscale (fluency of movements and absorption) scores asresponse variables containing summarized information about perceptual experiences of performers. In order to check for possible mediating or confounding effects of training parameters on the action-perception variables’ covariance, we included two additional variables which measured the degree of engagement of participants with the task. Results revealed that the temporal structure of fluctuations of the dominant leg, as measured by the Hurst exponent, was a strong mediator of effects of training variables and the subdominant leg fluctuations, on the flow scale and the subscale scores. The magnitude of Hurst exponents of both legs was informative about the degree of stability within the performer-environment system. The degree of critical slowing down, as measured by Hurst exponents, consistently co-varied with the flow scale and subscales. The experience of flow during the slackline walking task was dominantly saturated by the perceived fluency of movements and less so by the absorption experience. The stable co-variance of perception-action variables signified the embodied nature of the flow experience.

## Introduction

Flow has been historically investigated in sport and exercise for its association with exceptional performance (Jackson et al., 2001; Swann et al., 2018). Commonly defined as a harmonious psychological state, intrinsically rewarding, involving intense focus and absorption in a specific activity (Csikszentmihalyi, 1975, 2002; Swann et al., 2018; Stoll, 2019), flow has been contextualized in a framework of challenge-skill balance, clear goals and sense of control (Jackson and Csikszentmihalyi, 1999; Nakamura and Csikszentmihalyi, 2002). Under this view, the state of flow has been traditionally measured solely by subjective methods (Jackson and Eklund, 2002, 2004) without attempts to relate it *empirically* to behavioral measures. This is curious because in physical activities the state of flow was theoretically connected to the fluency of movements (Rheinberg et al., 2003; Engeser and Rheinberg, 2008), which obviously has a behavioral, action content. In this paper we make the first empirical attempt to reconcile this methodological and theoretical gap.

Optimal psychological experiences, underlying the excellence in performance, have been mainly related to flow or clutch states, typically experienced in contexts of achievement and pressure (Swann et al., 2017a, b). In contrast, flow has been generally described in contexts of exploration and flexible outcomes as well as experiences of enjoyment during the activity and lower perceived effort (Swann et al., 2019). As such, the relationship of flow with performance in exercise has been widely reported in the literature (Dietrich, 2004; Engeser and Rheinberg, 2008; Schüler and Brunner, 2009; Fernández et al., 2015; Ufer, 2017). In this line, the basis of flow has been mostly settled on psychological (Swann et al., 2017a, b; Stoll, 2019), physiological (Dietrich, 2004; Keller et al., 2011; Tozman et al., 2015) and psychophysiological factors (Swann et al., 2012). This research has found evidences of brain inhibition of self-reflective introspection during tasks, self-awareness reduction, focused attention and automatic actions among other effects (Jackson and Csikszentmihalyi, 1999; Nakamura and Csikszentmihalyi, 2002; Goldberg et al., 2006; Harris et al., 2017). Flow state has been also described through sensations like lack of weight, lack of fatigue, movement efficiency, and more integratively, as fusion with the environment (Fuentes-Kahal and del Cerro, 2012; Bertollo et al., 2016). In this line, the ecological psychology, and more concretely the ecological dynamics, explains the conscious mind as the very physical relation which emerges at the level of performer-environment system (Araújo et al., 2017). Consequently, phenomenological experiences cannot be understood simply embracing an organism-centered view (Davids and Araújo, 2010).

Within the framework of ecological dynamics, the performer and the environment are continuously integrated as the action regulation unfolds. As the perceptions of affordances (opportunities of action) contingently regulate the actions, and hence cognitions, actions reciprocally create new perceptions for prospective (future) actions. This action-perception cycle is crucial for understanding how conscious experiences emerge. The *informed awareness* is the information about oneself (e.g., proprioception, interoception) *in relation* to the environmental information (Shaw and Kinsella-Shaw, 2007). In this paper, we assume that it is this informed awareness that can reach the state of flow. According to the flow short scale (FSS) (Rheinberg and Vollmeyer, 2003; Rheinberg et al., 2003; Engeser and Rheinberg, 2008; Peifer et al., 2014), we approach the flow experience as consisting of two dimensions: (a) fluency of movements, i.e., sense of control, and (b) state of absorption by the activity when demands of tasks and skills are in balance (Sheldon et al., 2015). The informed awareness in the state of flow hence would incorporate the self-information of fluent and flexible control of the body-environment coupling and dominantly task goal focused attention. The state of *non-flow* would then be experienced as non-fluent, non-flexible and hence effortful control of the body-environment coupling with dominantly internal focus of attention.

Several types of specifying information are constitutive of the informed awareness (Shaw and Kinsella-Shaw, 2007), such as: information of performer’s needs, goals, effective means, the adaptiveness of enacting those means, as well as the progress toward the goal. These types of information are dynamically assembled in a form of a cycle consisting of continuous perception of and acting on affordances which have been defined as opportunities, invitations or solicitations for action (Gibson, 1979; Araújo et al., 2006; Withagen et al., 2012; Bruineberg and Rietveld, 2014), dwelling on many time scales. Every bodily action (performatory or exploratory) has a perceptual, and hence cognitive, role. Cognition is being constrained on-line by actions, and in this sense, bodily actions as well as the environment are constitutive to the cognition itself. In other words, informed awareness, as well as cognition as a part of it, is a distributed, embodied and thus, emergent property (Balagué et al., 2019) of the performer-environment system. In challenging^1^, non-trivial tasks, demanding high skillfulness, the larger the adaptiveness of dynamic assembling of these types of information (i.e., attunement to affordances), the higher the flow is. The adaptiveness of the dynamic assembling would *behaviorally* correspond to the *fluency of movements*, which is one of the two subscales of the FSS. This means that the fluency of movements is a necesary (but possibly not sufficient) for the experience of flow. Hence, within the ecological dynamics framework the fluency of movements as important component of flow can be defined as a locally^2^ optimal attunement (Bruineberg and Rietveld, 2014) to and acting on affordances during challenging tasks. That is, the behaviorally manifested fluency of movements would be a consequence of the attunement to the affordances. Consequently, the *subjective experience* of fluency of movements (sense of control), as a component of the informed awareness, would be a consequence of the attunement to affordances. This attunement to affordances would also stimulate the absorption component of the flow experience by maintaining a task goal instead of internally proprioceptive focused attention due to the effortful control.

As the informed awareness is a conscious experience, it follows that it is reportable, i.e., subject to verbal reports. This means that although informed awareness, *itself*, is not a process involving propositional content, it can be nevertheless socio-linguistically contextualized (by experimenters who linguistically engage participants) in such a way so that the performer can provide verbal reports^3^ *about* it. Hence, the data collected using the flow questionnaire can be defined as: judgment based time-delayed, coarse-grained verbal reports about the informed awareness experienced by performers during their engagement with the task. We use the word “coarse-grained” because while the informed awareness fluctuates at different time scales, the verbal reports must summarize that rich experience in a form of verbal statements. While the results of such questionnaires cannot be treated as a “gold standard” in determining behavioral or phenomenal experiential processes, it is also true that the concept of flow experience was and is still operationalized only in a form of questionnaire. In reaction to it, current literature is suggesting to offer more practical setting in collecting real-time data and capturing the dynamic nature of flow in exercise (Jackman et al., 2019).

**FIGURE 1:**
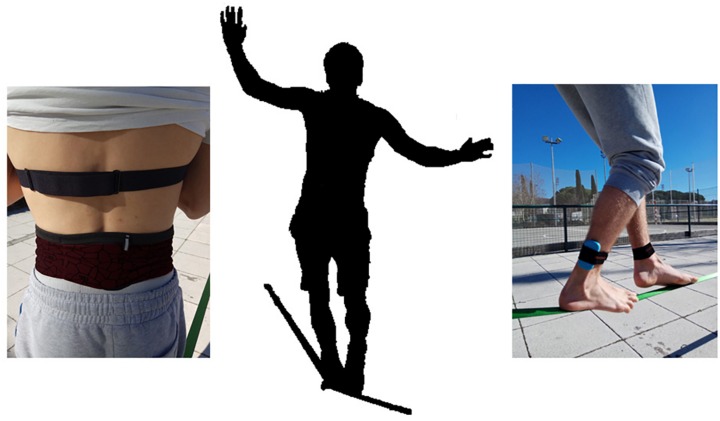
Slacklining with accelerometers placed in both ankles and the Center of Mass. Written informed consent was obtained from the depicted individuals for the publication of these images.

Then, of particular interest for our purposes was considering a task emerging from a strong performer-environment coupling, in which the actions of the performer change the state of the environment and vice versa, and which co-regulating cycle exists at more than one characteristic timescale. Slackline walking is one of such challenging tasks which, besides the important visual coupling, relies on the tight mechanical coupling of the performer with the slackline (Paoletti and Mahadevan, 2012). The goal of the task is to keep balancing the body in upward position on the slackline positioned at some height from the ground, and walk successfully some distance. Dynamically, it is an unstable inverted pendulum system with extremely narrow support base, which has to be stabilized by continuous perceptions-action cycles. This process of stabilization of an unstable system generates fluctuations. The body-slackline coupling is so strong that the fluctuations of the contact feet (body) are kinematically indistinguishable from the fluctuations of the slackline (environment) itself. However, if the slackline has, for example, different tension these fluctuations will change their character and the rest of the cognitive engagement will have to change as well. The cognitive engagement of the performer extends out of the body including the environment. Hence, kinematic fluctuations are in fact fluctuations of the whole performer-environment system rather than merely fluctuations of the performer or environment alone (Figure 1). This mechanical contact deforms the feet tissues and provides tactile and proprioceptive information for the state of coupling between the performer and the environment. The specifying haptic information enables the feeling of the limb positions and their changes relative to each other, relative to the body, and relative to the environment. The deformation of feet tissues, as well as other bodily components and their immediate response, due to their elastic-mechanic properties is the first level of movement and posture control. This control involves the body as direct regulator (inhibitor or activator) of the rest of relevant cognitive engagements in accomplishing the task. In this sense the cognition is embodied (Van de Laar and Regt, 2009). The task requires a continuous perceptual coupling of the performer with the slackline, and the rest of the environment, in order to trace minute changes in slackline-body fluctuations. This is necessary in order to avoid the enhancement of fluctuations through a positive feedback which would inevitably bring about a loss of stability of the center of mass (CoM) and task disengagement. In other words, performers continuously strive to stabilize the perception of walk-on-ability affordance by continuous adaptation of their body movements and perceptual systems at different time scales. Any deviation from this locally optimal attunement to the walk-ability affordance induces a tension that has to be reduced by additional action. This tension reduction is equivalent to stabilizing their CoM. As the slackline-body coupling shapes the external – and self-information by continuously regulating the actions of performer, the coupled body-slackline dynamics becomes constitutive of the informed awareness. The adaptiveness of this dynamic information assembling is reflected in fluency of movements of limbs and the CoM of the body. In other words, the smaller and smoother the deviations from the locally optimal attunement and needs of their reduction, the higher the flow experience of performers would be. In this sense, flow experience as constituent of the informed awareness arises and is maintained through the shared information (defined as co-varying processes) within the locally optimal perception-action cycles of performers. In other words, the complementary pair of flow experience and behavior may be formulated as a dynamic product of these co-varying processes that create the locally optimal perception-action cycles. Verbal reports are just propositions about these co-varying processes constituent of the informed awareness.

Previous research has shown that stability during standing posture and human locomotion in unsteady conditions is produced through adjustments of motor control strategies’ timing and compensatory synergies (Wang et al., 2014; Santuz et al., 2018). Particularly important study in this sense is Delignières et al. (2011) which convincingly argued about the velocity (and not position) dependent control of postural sway. These compensatory movements produce kinematic fluctuations around the task goal value, i.e., maintaining orthogonal (90°) and collinear to gravity force vector position of body with respect to the slackline support base, and maybe detected and classified according their stabilizing or destabilizing effects. Quickly suppressed deviations would tend to provide a better stabilizing control; in contrast, fluctuations that would positively add to the already extant deviation would tend, especially on longer time scales, to produce larger deviations from the task goal and a destabilizing effect. The temporal co-variation among the subsequent adjustments must be negative and produce anti-persistent or anti-correlated time variability. Otherwise, positively correlated adjustments or persistent properties would generate larger fluctuations from the task goal. That is, in anti-persistent fluctuations, deviations in one direction are statistically more likely followed by subsequent deviations in the opposite direction, and in persistent fluctuations deviations in one direction are statistically more likely to be followed by subsequent deviations in the same direction. Persistent fluctuations, signifying critical slowing down phenomenon and the impending instability (Scheffer et al., 2009), have been found in more rigid control during exercise, e.g., cases of extreme fatigue (Vázquez et al., 2016). On the other hand, anti-persistent fluctuations have been related to tighter, but rapid and flexible, control of kinematic variables (Terrier and Dériaz, 2012; Vázquez et al., 2016). Velocity-based control (with a transition from anti-persistence to persistence) have been shown in control of the postural sway (Boulet et al., 2010; Delignières et al., 2011). Moreover, some researchers have found relationships between experience, training, and skill levels with temporal co-variation of performance variables (Wijnants et al., 2009; Den Hartigh et al., 2015; Nourrit-Lucas et al., 2015). In particular, the postural and stride to stride control has been studied using the temporal variability of kinematic variables and quantified through Hurst (H) exponents (Balasubramaniam et al., 2000; Boulet et al., 2010; Delignières et al., 2011; Terrier and Dériaz, 2012; Vázquez et al., 2016).

Accordingly, based on what was discussed above, the aim of the current study was to capture the effects of the co-varying bi-directional process within the continuous perception-action cycle of a slackline walking task. Particularly, we were interested on how the dynamical stability of performance affects the embodied and extended information that shapes the flow experience in performers.

## Materials and Methods

### Participants

Nineteen volunteer Spanish slacklining practitioners (17 males, 2 females, 25.3 ± 4.9 yo, 70.21 ± 8.79 kg, 1.79 ± 0.05 m) from a faculty of Sport Sciences and a Slackline Club participated in the study. To be included in the sample they had to be able to perform the proposed slackline walking task (see section “Procedures”) and respond to the proposed flow scale questionnaire in English language (see section “Procedures”). Before starting, all participants completed a questionnaire to confirm their health status, their dominant/support leg (left, *n* = 16; right, *n* = 3) and subdominant leg, as well as an informed consent form. The experiment was approved by the local Research Ethics Committee.

### Procedures

All participants performed a continuous slackline walking task (Gibbon Slackline TM, ID Sports, Stuttgart, Germany) at a freely chosen velocity, without shoes and without falling during 30 s on a band of 10 m long and 5 cm width. They were not exposed to changes in their visual or acoustic information during the trials. They had a maximum of three attempts, separated by a maximum of 5 min rest to accomplish the task. The tension (T) of the slackline’s anchors (5.28 ± 0.65 kN), placed at 0.85 m from the ground, was calculated through the following formula:

T⁢(kN)=[L⁢(m)×W⁢(kg)]/[S⁢(m)×400]

where W is the weight of the participants, L is the length of the slackline (10 m) and S is the sag under load (ensuring at least 0.5 m in the center) (Conley, 2006).

The dynamic stability of the performer-slackline coupling was measured through the temporal variability of the acceleration fluctuations of the ankles, the body segments closer to the slackline, and the CoM, reflecting the postural control during the task. To this end, accelerometer devices WIMU PRO^TM^ (Real Track Systems, Almería, Spain) were placed and fixed with supports on both dominant and subdominant ankles, on the outside part above the lateral malleolus (Mannini et al., 2013), and in the CoM, placing the accelerometer on the zone of L3 (Moe-Nilssen and Helbostad, 2004; Schütte et al., 2016; see Figure 1). The acceleration was recorded at a sample frequency of 100 Hz to ensure enough data (>1024) to analyze through Detrended Fluctuation Analysis (DFA) a task lasting 30 s.

The English version of the Flow Short Scale (FSS) (Rheinberg et al., 2003; Engeser and Rheinberg, 2008), validated and applied experimentally to other flow studies (Engeser et al., 2005; Schüler, 2007; Schüler and Brunner, 2009), was administrated at the end of the task. All participants were previously familiarized with the FSS questionnaire. The flow experience (F_exp_) was measured in ten items (Cronbach’s α = 0.81) that were divided in two different subscales: the fluency of movements scale (F_mov_) including items 2, 4, 5, 7, 8, and 9, and the absorption by the activity scale (A) including items 1, 3, 6, and 10.

### Data Analysis

Time series for the CoM, the dominant and subdominant leg which were subject to analysis were Euclidean metrics of the 3D Cartesian acceleration components. The Euclidean metrics were directly provided by the accelerometers. The DFA was performed on the data series of acceleration and the Hurst (H) exponent was calculated to assess the temporal structure and time variability properties of the kinematic behavior (*N* = 3072 data points). DFA was conducted as follows (according to Peng et al., 1994, 1995; Ihlen, 2012): first, the total length of the acceleration time series (*N*) was integrated by using the following equation:

$$Y\,\left(i\right)\equiv \sum_{k=1}^{i}\left[x_{k}-x\right]$$

Where *x*_*k*_is the time series of acceleration and *x*is the average acceleration of the *N* data points. A quadratic polynomial function was then used to fit the time series to calculate the local trend (Ihlen, 2012). The resulting, i.e., velocity^4^, time series were divided into different windows scales *n* of equal length, with the local trend being subtracted in each window. The maximum scale of 512 data points was chosen according to Kantelhardt et al. (2002). For each window the root mean square (RMS) fluctuation was calculated by using the following equation:

$$\mathrm{RMS}=\sqrt{\frac{1}{N}\,\sum_{k=1}^{N}{\left[y\,\left(k\right)-y_{n}\,\left(k\right)\right]}^{2}}$$

Where the *y*(*k*) are the integrated (i.e., velocity) time series and the *y_n_(k)* is the local trend in each box. The H exponent, obtained as the slope value of the linear regression between the scale and local fluctuations on a log-log diffusion plot, was used to determine the temporal structure of the time series fluctuations.

H exponent values in the range 0 < H < 0.5 were associated with anti-persistent character of velocity fluctuations, while 0.5 < H ≤ 1 values were associated with their persistent profile (Delignières et al., 2011; Ihlen, 2012).

#### Shared Information Between Flow Scores, Training Frequency/Age and Time-Variability of Kinematic Behavior

In order to detect the degree of shared information between the flow scores (F_exp_, F_mov_, and A), the training (ω and τ) variables and behavioral variables (H_subdom_, H_dom_, H_CoM_), we conducted a Pearson correlation (*r*) and partial correlation analysis (ρ). Then, we performed a series of stepwise regression analyses in order to find out the best explanatory variable(s) responsible for the variance of flow experience scale F_exp_ and its subscales F_mov_ and A, which were treated as response variables.

##### Potential explanatory variables

ω = training frequency (hours per week): Mean = 2.29 ± 2.98; Min = 0.5; Max = 13
τ = training age (years of training): Mean = 3.05 ± 2.87; Min = 0.5; Max = 13
H_subdom_ = Hurst exponent of the subdominant leg
H_dom_ = Hurst exponent of the dominant leg
H_CoM_ = Hurst exponent of the center of the body mass

##### Response variables

F_exp_ = flow experience (full scale)
F_mov_ = fluency of movements (subscale)
A = absorption (subscale)

We checked the robustness of the stepwise regression results in three ways. First, we made series of standard simple and multiple regression procedures in order to control for possible confounding or mediating effects within the set of potential explanatory variables (Baron and Kenny, 1986). Second, we performed a series of forward and backward stepwise regression analysis and checked the level of congruence of results. Third, we applied a principal component analysis to highly correlated training variables (ω and τ), to construct a composite linear combination of both sets of standardized scores, in order to manipulate the number of degrees of freedom of the regression model, i.e., the number of potential explanatory variables. In the model including only directly measured variables, there were five potential explanatory variables, and in the model with the principal component there was one less, that is four potential explanatory variables. The variance explained by the explanatory variables was estimated by multiple coefficient of determination *R*^2^. We reported coefficients of multiple coefficient of determination (*R*^2^) adjusted to degrees of freedom of the model. Multiple regression effect sizes were expressed in Cohen’s *f*^2^. According to Cohen’s (1988) guidelines, *f*^2^ ≥ 0.02, *f*^2^ ≥ 0.15, and *f*^2^ ≥ 0.35 represent small, medium, and large effect sizes, respectively. Significance level was set on *p* < 0.05. Data analysis was conducted via Matlab© R2013b and Statistica 7 software packages.

## Results

**FIGURE 2:**
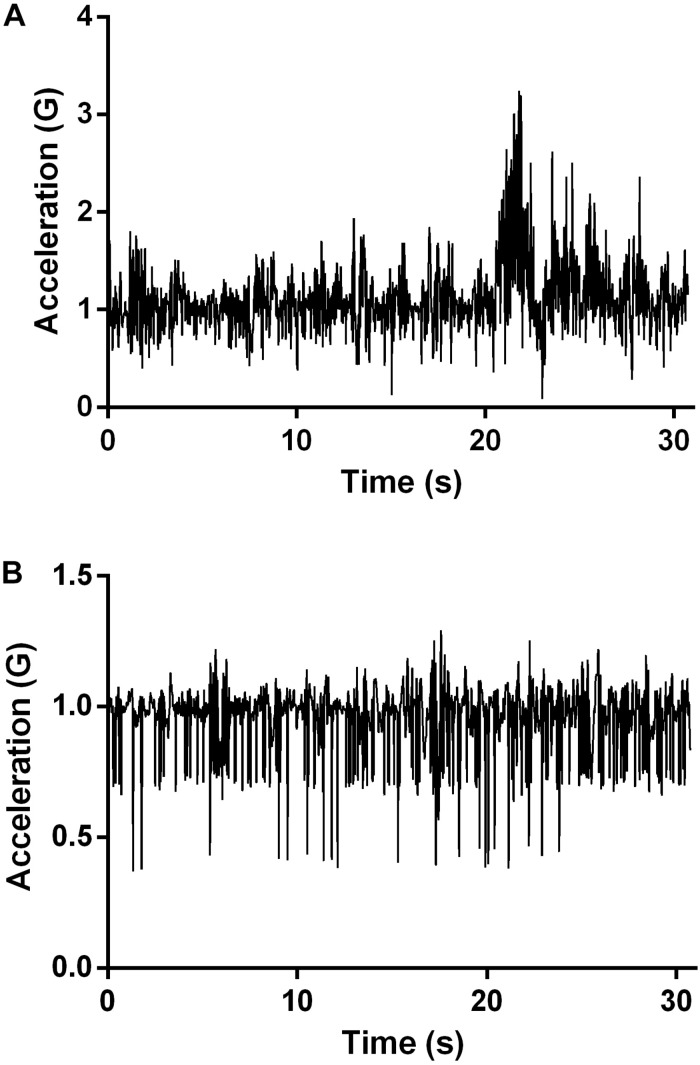
Examples of individual time series with **(A)** persistent (H = 0.89) and **(B)** anti-persistent (H = 0.41) fluctuation dynamics.

**FIGURE 3:**
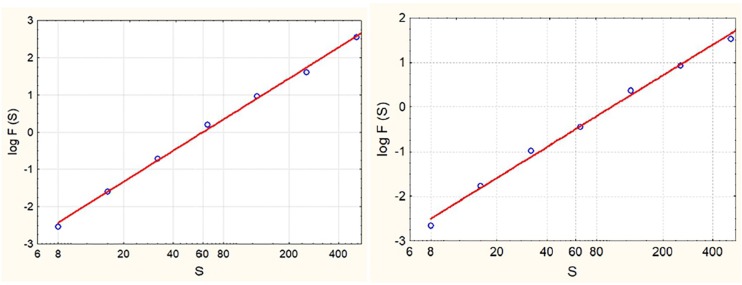
Typical diffusion plots for two participants. F(S) is the magnitude of fluctuations as measured by the RMS (see Eq. 2), and S is the scale.

The F_exp_ was rated considerably high (5.06 ± 0.89), as its two subscales (*F*_mov_ = 5.1 ± 1.17; *A* = 5.01 ± 0.76). The DFA analysis showed a persistent temporal structure of velocity fluctuations in both ankles (H_dom_ = 0.68 ± 0.11; H_subdom_ = 0.71 ± 0.11) and a weakly anti-persistent fluctuations of the CoM (H_CoM_ = 0.49 ± 0.05). Figure 2 shows two examples of individual time series with persistent and anti-persistent fluctuation dynamics, respectively. Cross-over of the slope, i.e., the Hurst exponent, was not detected (see Figure 3). The linear fit to diffusion plot data points in all 19 cases was statistically significant (*p*_min_ = 0.03, *p*_max_ = 0.001) and high (*R^2^* = 0.76 ± 0.02).

### Correlation Analysis of Potential Explanatory and Response Variables

Correlation analysis revealed several important clusters of relationship. F_exp_ showed quite strong association with the subscale F_mov_ (*r* = 0.96; *p* = 0.000001) and strong relationship with subscale A (*r* = 0.73; *p* = 0.0009). Moreover, H_subdom_ and H_dom_ were highly positively correlated (*r* = 0.77; *p* = 0.0001) while showing no correlation with H_CoM_ (*r* = 0.24; *p* < 0.235, and *r* = 0.19; *p* = 0.436), respectively. Also, flow subscales F_mov_ and A were moderately related (*r* = 0.50; *p* = 0.03). Training variables ω and τ showed strong association (*r* = 0.71; *p* = 0.001).

Training frequency (ω) was also moderately associated to most of other variables: F_exp_ (*r* = 0.56; *p* = 0.012); F_mov_ (*r* = 0.56; *p* = 0.012); H_subdom_ (*r* = −0.51; *p* = 0.026) and H_dom_ (*r* = −0.57; *p* = 0.01), while training age (τ) had significant medium relationship only with H_dom_ (*r* = −0.49; *p* = 0.034).

H_subdom_ and H_dom_ showed moderate to high associations with flow scale F_exp_: (*r* = −0.59; *p* = 0.008); (*r* = −0.72; *p* = 0.001), respectively, and its subscales F_mov_ (*r* = −0.55; *p* = 0.015); (*r* = −0.69; *p* = 0.001) and A (*r* = −0.46; *p* = 0.05); (*r* = −0.50; *p* = 0.029), respectively.

Controlling for joint effects of training variables ω and τ, the associations between the flow scale scores F_exp_ and its subscales F_mov_ (ρ = −0.93; *p* = 0.00001) and A (ρ = −0.69; *p* = 0.002) decreased. The association of flow scale F_exp_ and the F_mov_ subscale with the H_dom_ variable were maintained: ρ = −0.59; *p* = 0.013; ρ = −0.56; *p* = 0.019, respectively. Also, the statistically significant relationship between H_dom_ and H_subdom_ was maintained (ρ = 0.67; *p* = 0.03).

Controlling for H_dom_, however, removed the statistically significant associations between training variable ω and the flow scale F_exp_ (ρ = 0.27; *p* = 0.284), as well as its subscales F_mov_ (ρ = −0.28; *p* = 0.265) and A (ρ = −0.1; *p* = 0.694).

### Multiple Regression Analysis

In general, the results of the Baron and Kenny (1986) procedure revealed H_dom_ as a potential strong mediating variable. The results were sufficiently robust with respect to changes of the model seeking procedures (stepwise vs. backward) and the manipulation of the degrees of freedom of the model. The backward stepwise procedure revealed identical model to the one obtained by the forward stepwise procedure for the F_exp_ and F_mov_, but not for A scores. The model degrees of freedom manipulation showed qualitatively the same results, but the statistical significance of the model fit was larger than in forward stepwise regression. However, this model had higher Durbin-Watson statistic and that was the reason to proceed with the interpretation of the original stepwise regression results. Further, we present the results from the forward stepwise regression results noting that the results for absorption scale A have to be taken with more care since they were more fragile with respect to the model used.

#### Multiple Regression Analysis of Flow-Scale Scores

Tolerance scores of explanatory variables that entered the forward stepwise regression equation, i.e., H_dom_ and ω, were at satisfactory level (*T* = 0.67, *T* = 0.67), respectively. Durbin-Watson statistic (*DW* = 2.60) revealed an independence of residual values. Residuals were also normally distributed (Shapiro-Wilk *p* = 0.294) and satisfied the criterion of homoscedasticity. Cooke’s Distance statistic (*D*_median_ = 0.03; *D*_max_ = 0.27; *D*_min_ = 0.000001) showed that there were no influential cases potentially biasing the results.

Multiple correlation between the system of explanatory variables H_dom_, ω and the F_exp_ scores was statistically significant and strong: *R* = 0.74; *R*^2^ = 0.49; *F*(2, 16) = 9.63; *p* < 0.002. Cohen’s *f*^2^ = 0.96 revealed a very large effect size. Partial regression coefficient was significant only for H_dom_ (β = −0.58; *t*(16) = −2.84; *p* < 0.01), but for ω showed no statistically significance (β = −0.23; *t*(16) = 1.11; *p* < 0.284).

#### Multiple Regression Analysis of Fluency of Movements Subscale Scores

Tolerance scores of explanatory variables who entered the forward stepwise regression equation H_dom_ and ω were (*T* = 0.67, *T* = 0.67), respectively. Durbin-Watson statistic (*DW* = 2.68) revealed an independence of residual values. Residuals were also normally distributed (Shapiro-Wilk *p* = 0.76) and satisfied the criterion of homoscedasticity. Cooke’s Distance statistic (*D*_median_ = 0.03; *D*_max_ = 0.14; *D*_min_ = 0.002) showed that there were no influential outliers potentially biasing the results.

Multiple correlation between the system of explanatory variables H_dom_, ω and the F_mov_ scores as response variable was statistically significant and strong: *R* = 0.72; *R*^2^ = 0.46; *F*(2, 16) = 8.68; *p* < 0.003; *f*^2^ = 0.85 signified a very large effect size. However, partial regression was significant only for H_dom_ (β = −0.553; *t*(16) = −2.61; *p* < 0.019), and showed no statistical significance for ω (β = −0.245; *t*(16) = 1.16; *p* < 0.26).

#### Multiple Regression Analysis of Absorption Subscale Scores

Tolerance scores of explanatory variables which entered the regression equation: H_dom_ and H_CoM_ were (*T* = 0.94, *T* = 0.94), respectively. Durbin-Watson statistic (*DW* = 2.07) revealed the independence of residual values. Residuals were also normally distributed (Shapiro-Wilk *p* = 0.28) and satisfied the criterion of homoscedasticity. Cooke’s Distance statistic (*D*_median_ = 0.014; *D*_max_ = 0.86; *D*_min_ = 0.0002) showed that there were no influential cases potentially biasing the results.

Multiple correlation between the system of explanatory variables H_dom_, H_CoM_ and the A scores as response variable was statistically significant and of medium strength: *R* = 0.58; *R*^2^ = 0.25; *F*(2,16) = 4.00; *p* < 0.039. Nevertheless, Cohen’s test (*f*^2^ = 0.33) revealed medium effect size. However, partial contributions of both variables were not significant, although H_dom_ was a borderline case: (β = −0.43; *t*(16) = −2.05; *p* < 0.057) and (β = −0.297; *t*(16) = −1.41; *p* < 0.177).

## Discussion

The aim of the current study was to capture the effects of correspondence within the bi-directional continuous perception-action cycle of a slackline walking task. The correlation analysis showed some intriguing relations between the treated variables. F_exp_ was dominantly associated to the fluency of movements subscale F_mov_ and less to absorption subscale A. After controlling for effects of training variables ω and τ and action variable H_dom_, the differences of these associations increased. Moreover, controlling for training variables maintained the statistically significant association between the action variable H_dom_ with the flow scale F_exp_ and its fluency subscale F_mov_, while controlling for H_dom_, removed the significant associations between training variables ω and τ and the flow scale F_exp_ and its subscales F_mov_ and A. This may mean that it is the *information related to action* (i.e., the pragmatic information) which is the main constituent of the informed awareness of flow experience for this task. The regression analysis also consistently revealed H_dom_ as an explanatory^5^ variable of the flow experience (the full scale and its subscales), although the absorption subscale A showed larger part of specific variance in comparison with the fluency of movements subscale F_mov_. Absorption refers to a task engagement with minimal self-consciousness when demands and skills are in balance (Rheinberg and Vollmeyer, 2003; Peifer et al., 2014). The constraints of the slackline walking task may have not been able to induce such type of dominant outward focused attention in performers. It is probable that the slackline task is, to a certain degree, demanding of self-internally focused attention. Focusing on the body-slackline mechanical contact, i.e., enabling crisp self-information from tactile and proprioceptive sources (Shaw and Kinsella-Shaw, 2007), may be crucial for accomplishing the task.

Indeed, slackline walk is commonly used as a meditative (mindfulness) practice (Curtis and Braga, 2018) which requires increased self-awareness. On the other hand, the flow experience, by its definition, requires reduced self-consciousness (Sheldon et al., 2015). It may be that there was a trade-off relation between these conflicting requirements for absorption, responsible for the results obtained in this specific task. This finding calls to attention to the possibility of varying degrees of involvement of movement fluency (sense of control) and absorption processes for the flow experience in different tasks.

Taking together these results with those of the correlation analysis, we can go a step further claiming that perception-action-environment processes, responsible for determining the values of H_dom_, have a causal role in forming a flow experience, as constitutive of the informed awareness of participants. In this case H_dom_ would play a role of a nearly full mediator variable between the rest of explanatory variables and the response variables (flow scores). Neither training age nor frequency of training significantly predicted the flow scores (subscales and the full scale). Their effects, as well as the effect of H_subdom_ on the flow experience, were clearly mediated by the H_dom_ variable. The temporal structure of fluctuations of the whole coupled body-environment (i.e., slackline) system, as measured by H_dom_, co-varies with and corresponds to the states of informed awareness (particularly the perception of fluency of movements). Note that these processes are not *only* neurologically regulated and even less they are skull bound. In other words, the changes in body-slackline coupling, i.e., the body-environment dynamics, as measured by H_dom_, regulate the flow experience. Any deviation from the locally optimal attunement to the walk-on-ability-affordance creates a state that has to be stabilized by compensatory actions. Compensatory actions are a result of online explorations of stability. There is hardly a pre-set value, a mental representation of a “correct” action or action sequence that can be applied. A pre-set correct action simply cannot exist because there is an ever changing unpredictable flow of the performer-slackline interactions. What is important is to locally solve the perceptual attunement and action on the walk-on-ability affordance. Hence, all perceptual explorations and compensatory movements are made “on the fly” as a result of a successful contingent perception-action explorations. If these compensatory perception-action cycles come close to bring about instability in the performer-environment coupling they destroy the flow awareness and vice versa. Thus, flow and particularly the fluency of movements experience does not correspond to “*automaticity*”^6^ of actions (see Harris et al., 2017 for opposite opinion), but to their functional flexibility, i.e., *adaptability*. Note again that the flow experience, as a part of the informed awareness *itself*, does not have to contain any propositional properties, but nonetheless can, to a degree, be captured by linguistically engaged performers, using propositional statements given in the questionnaire. In this sense, the flow experience *itself* can be defined not as merely skull bound mental state, but as embodied and extended active process of informed awareness emerging at the level of performer-environment system.

While the velocity fluctuations of the ankles showed persistent fluctuations, the velocity fluctuations of the CoM showed dominantly anti-persistent behavior. The persistent dynamics reflects a more balanced interaction of negative and positive feedback loops within the system, and the anti-persistent dynamics a dominance of the negative-stabilizing feedback (Cuomo et al., 2000; Vázquez et al., 2016). The persistent time variability structure of the ankle’s velocity fluctuations reflected an exploratory and compensatory synergy of lower limbs to regulate the balance of the CoM on the webbing. In contrast, the anti-persistence of the CoM fluctuations signified its tightly controlled stability that has been enabled by different compensatory synergies reflected in ankle fluctuations (Latash et al., 2007; Singh et al., 2018). In general, it seems that the lower limbs explore possible coordination in order to form a negative feedback for the efficient positional control of the performer’s CoM. This points to the possibility that performers experiencing low flow need larger movement explorations (leg excursions) to acquire a functional control of the body’s CoM. On the contrary, high flow performers need less exploratory actions (less leg excursions) to attain the control. These differences signified variations in embodied cognitive strategies of performers while negotiating task constraints. The increased positive correlation of increments in the time series has been formally connected to the phenomenon of critical slowing down, i.e., the impending instability of the complex system, indicating the loss of system’s resilience (Scheffer et al., 2012). Thus, higher H values meant that, on average, positive serial correlations were larger and thereby there was a more emphasized critical slowing in the system (Vázquez et al., 2016). This means that the performer-slackline system of participants with higher H values was less stable (i.e., increased the chances of critical transition, fall or task disengagement) than those with lower H values.

On the other hand, even a novice with several months of training may become well adapted to the constraints of the current task and release the attentional resources partly outward. One may hypothesize that a larger height or lower tension of the slackline, or both, would form a task with higher level of functional difficulty. Suitably modified task constraints may induce more direct effects of the training age and frequency of training on flow experience. Such task constraints may particularly affect effects of behavioral movement action variables such as H_dom_ on the absorption subscale scores due to larger salience of task internal related focus of attention in more challenging situations tasks of this type. These hypotheses warrant further investigation on a larger sample of performers.

The adaptability to environmental changes characterizes successful performers, because their system’s stability is reflected in their capacity to negotiate the induced perturbations through stable but flexible coordinated movements (Davids et al., 2012; Santuz et al., 2018). Even good performers need compensatory movement variability (Davids et al., 2012). Such performance conditions involve a coordinated action to integrate functionally the degrees of freedom of the performer-environment system (Davids et al., 2008). This integration is dynamically formed by the reciprocal interaction among the slower and shorter time-scale control loops of the performer-environment system (Hristovski and Balagué, 2010; Vázquez et al., 2016).

As slackline is not a regulated and competitive activity that performers practice regularly, the participants in this study could not be classified according their performance results, as was done in previous research (Den Hartigh et al., 2015). Regarding the retrospective self-report method used in this study, limitations should be also taken into account. Due to it, more research is warranted to confirm the current results. Moreover, a research based on bi- or multivariate time series of flow experience scores and behavioral quantities may provide in future more detailed and more realistic understanding of the continuous dynamic entanglement of processes which form the experience-behavior complementary pair.

In conclusion, as a first step toward the goals expressed in the previous passage, the stable co-variance of perception-action variables signified the embodied nature of the flow experience. The dynamic signatures of the whole performer-environment system, such as the critical slowing down, strongly affected the flow experience. In this sense, it is the ecological dynamics of the whole performer-environment system and the fluctuating dynamics of the continuous multi timescale perception-action cycles within it, that characterizes the experience of flow as a state of the informed awareness (Shaw and Kinsella-Shaw, 2007).

## Data Availability Statement

The datasets generated for this study are available on request to the corresponding author.

## Ethics Statement

The studies involving human participants were reviewed and approved by the Comité d’Ètica d’Investigacions Cliniques de l’Administració de Catalunya. The patients/participants provided their written informed consent to participate in this study.

## Author Contributions

NB, RH, LR, and LM conceived and designed the experiments. LM, LR, and NB performed the experiments. LM, PV, and RH analyzed the data. RH, LM, PV, and NB interpreting the results. LM, LR, PV, and NB contributed the reagents, materials, and analysis tools. LM, PV, NB, RH, and LR wrote the manuscript.

## Conflict of Interest

The authors declare that the research was conducted in the absence of any commercial or financial relationships that could be construed as a potential conflict of interest.

## Footnotes

1 Degree of challenge can be defined as a relation between the nominal task difficulty and the skill level of the performer.

2 Locally optimal – here means that there exist performer-environment configurations which temporarily and contingently enable the maximally possible fluency of perception – action cycle of the performer. This statement does not entail existence of mental representation with content in a form of e.g., pre-set optimal movement execution sequence.

3 Although uncommon, verbal reports have been used in ecological psychology. For example, the classical work of Warren (1984) used verbally reported judgments about affordances, i.e., informed awareness, in a stair-climbing task.

4 Although original time series were acceleration time series, by integrating we obtained the velocity time series. Velocity time series’ Hurst exponents (H_v_) differ from the Hurst exponents of acceleration time series (H_a_) by an additive constant: H_v_ = H_a_ + 1. Velocity time series, thus, belong to the Brownian motion type (H_v_ > 1) and acceleration time series to a noise type (0 < H_a_ < 1) of fluctuations (Delignières et al., 2011). However, because they differ only by additive constant, the meaning of the results is equivalent for acceleration and velocity profiles.

5 For both subscales and the full scale the unexplained, i.e., flow unique variance, was probably due to individual history differences, unaccounted specific constraints impinging on the perception-action system (Balagué et al., 2019), as well as the error variance.

6 Automaticity, on the other hand, may be attained in stable, attentionally non-demanding environments. It does not require a strong action-related attention focus and may allocate the attention resources to task unrelated thoughts (e.g., Balagué et al., 2012).
